# Comparative chloroplast genomics of 34 species in subtribe Swertiinae (Gentianaceae) with implications for its phylogeny

**DOI:** 10.1186/s12870-023-04183-1

**Published:** 2023-03-28

**Authors:** Lucun Yang, Shengxue Deng, Yongqing Zhu, Qilin Da

**Affiliations:** 1 Northwest Institute of Plateau Biology, Chinese Academy of Sciences, Xining, 810008 China; 2 Key Laboratory of Tibetan Medicine Research, Chinese Academy of Sciences, Xining, 81008 China; 3 Qinghai Key Laboratory of Qinghai-Tibet Plateau Biological Resource, Northwest Institute of Plateau Biology, Chinese Academy of Sciences, Xining, 810008 China; 4 Qinghai Environmental Science Research and Design Institute Co. Ltd, Xining, 810007 China; 5 Maqin County Forestry and Grassland Station, Maqin, 814000 China; 6 Bureau of Forestry in Hualong County, Hualong, 810900 China

**Keywords:** Chloroplast genomes, Single sequence repeat, Positive selection

## Abstract

**Background:**

Subtribe Swertiinae, a medicinally significant and highly speciose Subtribe of family Gentianaceae. Despite previous extensive studies based on both morphology and molecular data, intergeneric and infrageneric relationships within subtribe Swertiinae remain controversial.

**Methods:**

Here, we employed four newly generated *Swertia* chloroplast genomes with thirty other published genomes to elucidate their genomic characteristics.

**Results:**

The 34 chloroplast genomes were small and ranged in size from 149,036 to 154,365 bp, each comprising two inverted repeat regions (size range 25,069–26,126 bp) that separated large single-copy (80,432–84,153 bp) and small single-copy (17,887–18,47 bp) regions, and all the chloroplast genomes showed similar gene orders, contents, and structures. These chloroplast genomes contained 129–134 genes each, including 84–89 protein-coding genes, 37 tRNAs, and 8 rRNAs. The chloroplast genomes of subtribe Swertiinae appeared to have lost some genes, such as *rpl33*, *rpl2* and *ycf15* genes. Comparative analyses revealed that two mutation hotspot regions (*accD-psaI* and *ycf1*) could serve as effective molecular markers for further phylogenetic analyses and species identification in subtribe Swertiinae. Positive selection analyses showed that two genes (*ccsA* and *psbB*) had high Ka/Ks ratios, indicating that chloroplast genes may have undergone positive selection in their evolutionary history. Phylogenetic analysis showed that the 34 subtribe Swertiinae species formed a monophyletic clade, with *Veratrilla*, *Gentianopsis* and *Pterygocalyx* located at the base of the phylogenetic tree. Some genera of this subtribe, however, were not monophyletic, including *Swertia*, *Gentianopsis*, *Lomatogonium*, *Halenia*, *Veratrilla* and *Gentianopsis*. In addition, our molecular phylogeny was consistent with taxonomic classification of subtribe Swertiinae in the Roate group and Tubular group. The results of molecular dating showed that the divergence between subtrib Gentianinae and subtrib Swertiinae was estimated to occur in 33.68 Ma. Roate group and Tubular group in subtribe Swertiinae approximately diverged in 25.17 Ma.

**Conclusion:**

Overall, our study highlighted the taxonomic utility of chloroplast genomes in subtribe Swertiinae, and the genetic markers identified here will facilitate future studies on the evolution, conservation, population genetics, and phylogeography of subtribe Swertiinae species.

**Supplementary Information:**

The online version contains supplementary material available at 10.1186/s12870-023-04183-1.

## Introduction

Subtribe Swertiinae, a medicinally significant and highly speciose Subtribe of family Gentianaceae, includes approximately 539–565 species, which are distributed in alpine, temperate and alpine regions around the world but rarely in tropical and subtropical regions at low latitudes. East Asia and North America are the centers of diversification of this subtribe, with 137 species of 11 genera in China [1]. Many species of subtribe Swertiinae, such as *Halenia elliptica* D.Don, *Comastoma pedunculatum* (Royle ex G.Don) Holub., *Gentianopsis paludosa* (Hook.f.) Ma., *Lomatogonium carinthiacum* (Wulfen) Rchb., *Swertia mussotii* Franch. and *S. franchetiana* Harry Sm., are the original traditional Tibetan folk medicinal species called “Dida” (Zangyinchen). These species have been used to cure bilious hepatitis, cholecystitis, hepatogenous jaundice, and hypoglycemia diseases in China for a long time [2, 3]. Therefore, increasing attention has been given to the plants of subtribe Swertiinae due to their extensive pharmacological effects.

A robust and well-resolved phylogeny in subtribe Swertiinae is fundamental to understanding the evolution and diversification of this subtribe, including individual traits, species diversification and conservation. Over the past decades, growing evidence of molecular markers (such as plastid markers and nuclear ribosomal DNA) has greatly advanced our understanding of subtribe Swertiinae relationships [4–8]. The results have shown that some genera of subtribe Swertiinae are not monophyletic groups, including *Swertia*, *Gentianella*, *Comastoma*, and *Lomatogonium*. In particular, *Swertia* is strongly paraphyletic to other genera. Meanwhile, the phylogenetic relationships between genera and within genera of subtribe Swertiinae obtained using different molecular markers (fewer fragments of chloroplast DNA sequences and ITS sequences) were also inconsistent [4–8]. For example, the molecular phylogeny of Swertiinae based on *ITS* and *mat*K sequence data showed that the topmost part of the phylogenetic tree is a clade composed of *Megacodon, Gentianella*, *Swertia*, *Jaeschke*, *Lomatogonium*, and *Comastoma*, while the molecular phylogeny of Swertiinae based on *matK* and *rbcL* sequence data showed that the topmost part of the phylogenetic tree is a clade composed of some species of subgen. *Ophelia* of *Swertia* and, 7 species and varieties of the *Gentianella*. In addition, studies on anatomy [9–11], cytobiological chromosome numbers [12], pollen morphology [13], seed coat micromorphology [14–16] and other characteristics could not provide evidence for the definition of subtribe Swertiinae groups, showing that *Swertia*, *Gentianella*, and *Lomatogonium* are polyphyletic or paraphyletic genera. Therefore, effective methods for resolving phylogenetic relationships and evaluating previous classification of subtribe Swertiinae species are urgently needed.

Due to their moderate size, moderate nucleotide substitution rates and freedom from the problems of paralogy [17], plastid DNA sequences have been widely used for the reconstruction of plant phylogenies. A plastome phylogenomics approach has been successfully used to solve many enigmatic relationships within angiosperms and across all green plants [18–24]. Some studies have also revealed that chloroplast genome resources could offer helpful data for inferring the evolutionary relationships of Gentianaceae plants, thus reflecting vital evidence for a well-supported hypothesis of classification [25, 26]. To date, 34 complete chloroplast genomes of subtribe Swertiinae species have been sequenced, and some studies have been conducted based on these chloroplast genomes [25–28]. These studies initially probed the structural patterns of *Swertia* plastomes and the phylogenetic relationships within *Swertia*. Meanwhile, the phylogenetic relationships of subtribe Swertiinae were also examined. However, the phylogenetic relationships of subtribe Swertiinae obtained from the same chloroplast genome were inconsistent [25, 26]. Moreover, the structural patterns among genera within subtribe Swertiinae are still unknown. Thus, more evidence is crucial for further investigation.

The comparative analyses of the complete chloroplast genome structures among closely related species have yielded a wealth of information for taxonomically or phylogenetically recalcitrant lineages [29], such as Orychophragmus [30, 31], Epimedium [32], and Orchids [33]. In this study, we sequenced and reported the complete chloroplast genome of four *Swertia* species and conducted comparative chloroplast genomics with published chloroplast genomes of 30 other subtribe Swertiinae species. The study objectives were to (1) compare the sequences, structures, and gene organization of chloroplast genomes within subtribe Swertiinae; (2) identify polymorphic loci for future phylogenetic inference of the subtribe Swertiinae; and (3) elucidate the molecular evolution and phylogenetic relationships of subtribe Swertiinae.

## Results

### Structural features of subtribe Swertiinae chloroplast genomes

**Fig. 1:**
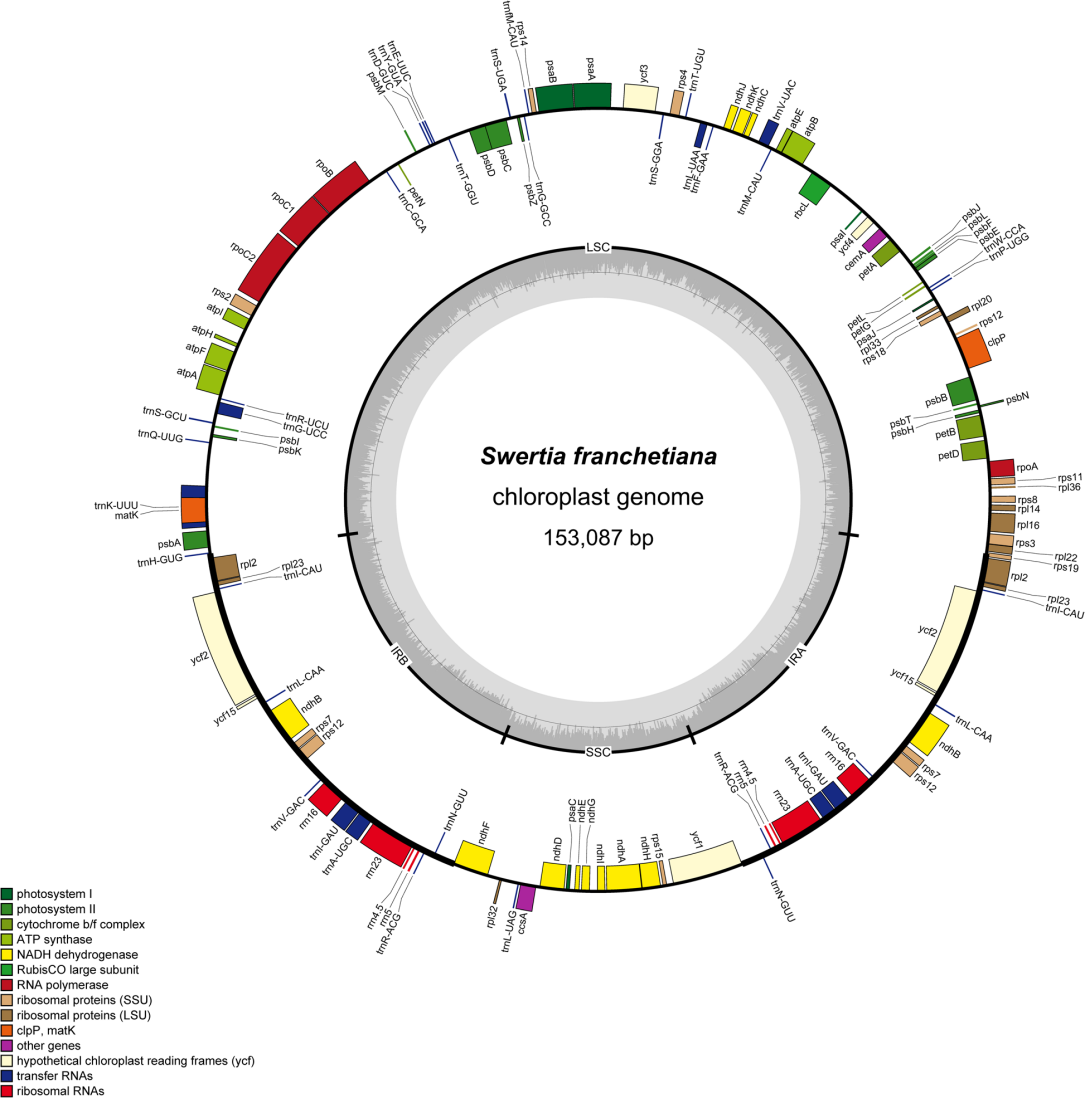
Structure and characteristics of the complete chloroplast genomes of 34 Subtribe Swertiinae species. Genes inside and outside the circle are transcribed clockwise and counterclockwise separately. Darker and lighter grey in the inner circle each represent GC and AT content

In this study, we analyzed the chloroplast genome features and gene contents of 34 species in 9 genera from subtribe Swertiinae (Table 1 and Table S1). All 34 chloroplast genomes displayed a typical quadripartite structure that was similar to that of the majority of angiosperm chloroplast genomes (Fig. 1). The length of the chloroplast genome of the 34 species varied between genera and species. The chloroplast genome length ranged from 149,036 (*S. pubescens* Franch*.*) to 154,365 bp (*Pterygocalyx volubilis* Maxim.), with an average length of 152,274 bp (Table 1). The longest chloroplast genome (154,365 bp) differed from the others by 0.614–5.329 kb. All complete chloroplast genomes were made up of four parts, namely, an LSC region (80,432–84,153 bp), an SSC region (17,887–18,476 bp), and two IR regions (25,069–26,126 bp). The GC content of the 34 species was very similar in both the whole chloroplast genome (37.5–38.26%) and the corresponding regions (LSC [32.18–36.36%], SSC [30.39%-33.66%], and IR [42.16%-43.38%]), with the IR regions having the highest GC contents (Table 1).

**Table 1:** The complete genome features of 34 species of 9 genera in *Subtribe Swertiinae*

Species	All length (bp)	GC (%)	LSC Length (bp)	GC (%)	SSC Length (bp)	GC (%)	IR Length (bp)	GC (%)	Gene number	tRNA gene number	rRNA gene number	Protein-coding gene
*Comastoma falcatum*	151,423	38.26	81,721	36.34	18,248	31.78	25,727	43.59	132	37	8	87
*Comastoma pulmonarium*	151,595	38.25	81,919	36.30	18,280	31.79	25,698	43.69	130	37	8	85
*Gentianopsis barbata*	151,123	37.85	82,690	35.80	17,887	31.77	25,273	43.34	131	37	8	86
*Gentianopsis grandis*	151,271	37.87	82,572	35.81	17,907	31.76	25,396	43.27	134	37	8	89
*Gentianopsis paludosa*	151,568	37.84	82,834	35.76	17,928	31.77	25,403	43.35	129	37	8	84
*Lomatogoniopsis alpina*	150,986	38.13	81,302	36.22	18,180	31.35	25,752	43.54	131	37	8	86
*Lomatogonium perenne*	151,678	38.16	81,979	36.28	18,237	31.46	25,731	43.52	131	37	8	86
*Pterygocalyx volubilis*	154,365	37.87	84,033	35.87	18,476	31.65	25,928	43.34	131	37	8	86
*Veratrilla baillonii*	151,962	38.24	82,475	36.35	17,983	30.39	25,752	43.44	132	37	8	87
*Halenia coreana*	153,198	38.22	83,252	36.36	18,372	32.16	25,787	43.39	134	37	8	89
*Halenia elliptica*	153,305	38.15	82,767	36.26	18,286	32.02	26,126	43.29	133	37	8	88
*Swertia bifolia*	153,242	38.06	83,496	36.16	18,200	31.89	25,773	43.33	133	37	8	88
*Swertia bimaculata*	153,751	38.03	84,156	36.02	18,089	32.07	25,753	43.39	134	37	8	89
*Swertia cincta*	149,089	38.20	80,481	36.34	17,946	31.79	25,331	43.42	133	37	8	88
*Swertia cordata*	153,429	38.05	83,612	36.16	18,037	31.75	25,890	43.3	133	37	8	88
*Swertia dichotoma*	152,977	37.50	83,044	35.55	18,303	31.25	25,815	43.02	132	37	8	87
*Swertia dilatata*	150,057	38.17	81,310	36.28	17,887	31.79	25,430	43.42	132	37	8	87
*Swertia diluta*	153,691	38.10	83,859	36.20	18,300	31.9	25,766	43.5	134	37	8	89
*Swertia erythrosticta*	153,039	38.10	83,372	36.18	18,249	31.89	25,709	43.33	133	37	8	88
*Swertia franchetiana*	153,428	38.20	83,564	34.66	18,342	33.22	25, 749	43.28	133	37	8	88
*Swertia hispidicalyx*	149,488	38.19	80,727	36.30	17,903	31.81	25,429	43.42	133	37	8	88
*Swertia kouitchensis*	153,475	38.15	83,595	36.23	18,348	31.93	25,766	43.47	133	37	8	88
*Swertia leducii*	153,015	38.17	83,048	36.35	18,395	31.90	25,785	43.44	134	37	8	89
*Swertia macrosperma*	152,737	38.22	83,046	36.31	18,231	31.99	25,730	43.50	133	37	8	88
*Swertia multicaulis*	152,190	38.10	82,893	36.25	18,343	31.82	25,477	43.35	131	37	8	86
*Swertia mussotii*	153,499	38.16	83,591	36.23	18,336	31.95	25,761	43.50	134	37	8	89
*Swertia nervosa*	153,690	38.12	83,864	36.25	18,254	31.82	25,786	43.37	131	37	8	86
*Swertia przewalskii*	151,079	38.1	81,780	33.22	18,193	33.66	25,553	42.16	133	37	8	88
*Swertia pubescens*	149,036	38.19	80,432	36.33	17,936	31.81	25,334	43.42	133	37	8	88
*Swertia punicea*	153,448	38.15	83,535	36.25	18,345	31.88	25,784	43.47	133	37	8	88
*Swertia souliei*	152,804	38.08	83,195	36.17	18,105	31.89	25,752	43.33	134	37	8	89
*Swertia tetraptera*	152,787	38.1	83,177	32.18	18,305	32.18	25,679	44.38	134	37	8	89
*Swertia verticillifolia*	151,682	38.14	82,623	36.26	18,335	31.83	25,362	43.48	134	37	8	89
*Swertia wolfgangiana*	153,225	38.06	83,528	36.17	18,219	31.88	25,739	43.34	134	37	8	89

The chloroplast genome gene contents of the 34 species showed slight variation. The gene contents ranged from 129 (*Gp. paludosa*) to 134 (*Gp. grandis* (Harry Sm.) Ma, *Veratrilla baillonii* Franch, *H. coreana* S.M.Han, H.Won & C.E.Lim, *S. bimaculata* Hook.f. & Thomson ex C.B.Clarke, *S. diluta* (Turcz.) Zuev, *S. leducii* Franch., *S. mussotii*, *S. souliei* Burk., *S. tetraptera* Maxim*.*, *S. verticillifolia* T.N.Ho & S.W.Liu and *S. wolfgangiana* Grüning) (Table 1). Accordingly, the number of protein-coding genes also varied, ranging from 84 to 89. However, the numbers of tRNA genes (37) and rRNA genes (8) were relatively conserved among species (Table S1). Among these protein-coding genes, four pseudogenes (*rps16*, *infA*, *ycf1* and *rps19*) were found. Except for the lack of the *rpl33* gene in the chloroplast genomes of* S. dilatate* C.B.Clarke, *S. hispidicalyx* Burkill, *P. volubilis* and *C. pulmonarium* (Turcz.) Toyokuni, the *rpl2* gene in the chloroplast genome of *C. falcatum* (Turcz.) Toyokuni and the *ycf15* gene in the chloroplast genome of *G. paludosa*, gene content differences were caused by the four pseudogenes. For example, due to the lack of the *rps16*, *ycf1* and *rps19* pseudogenes, the chloroplast genome of *Lomatogoniopsis alpina* T.N.Ho & S.W.Liu contained 131 genes (Table S1). Among all the genes, 18 genes (*trnK-UUU*, *rps16*, *trnG-UCC*, *atpF*, *rpoC1*, *ycf3*, *trnL-UAA*, *trnV-UAC*, *rps12*, *clpP*, *petB*, *petD*, *rpl16*, *rpl2*, *ndhB*, *trnI-GAU*, *trnA-UGC* and *ndhA*) in *H. elliptica*, *V. baillonii* and *S. punicea* Hemsl. contained only one intron, while 17 genes (*rps16* was absent or did not contain an intron) in the remaining 31 species of subtribe Swertiinae contained one intron. Two protein-coding genes (*ycf3* and *clpP*) in the chloroplast genomes of all 34 species contained two introns (Table S2).

The functions of major genes in the chloroplast genome of subtribe Swertiinae could be roughly divided into three categories (Table 2): photosynthesis, chloroplast self-replication and other. Genes associated with photosynthesis and self-replication made up the majority of the chloroplast genome.

**Table 2:** Gene composition of chloroplast genome of 34 species of 9 genus in Subtribe Swertiinae

Categroy	Group of genes	Name of genes
**Photosynthesis**	Photosystem I	*psa*A, *psa*B, *psa*C, *psa*I, *psa*J
	Photosystem II	*psb*A, *psb*B, *psb*C, *psb*D, *psb*E, *psb*F,*psb*H, *psb*I, *psb*J, *psb*K, *psb*L, *psb*M,*psb*N, *psb*T, *psb*Z
	NADH dehydrogenase	*ndh*A^a^, *ndh*B^a^, *ndh*C, *ndh*D, *ndh*E, *ndh*F,*ndh*G, *ndh*H, *ndh*I, *ndh*J,*ndh*K
	Cytochrome b/f complex	*pet*A, *pe*tB^a^, *pet*D^a^, *pet*G, *pet*L, *pet*N
	ATP synthase	*atp*A, *atp*B, *atp*E, *atp*F^a^, *atp*H, *atp*I
**Self-replication**	Ribosomal proteins (SSU)	*rps*2, *rps*3, *rps*4, *rps*7, *rps*8, *rps*11, *rps*12^c^, *rps*14, *rps*15, *rps*16^a^, *rps*18, *rps*19
	Ribosomal proteins (LSU)	*rpl*2^a^, *rpl*14, *rpl*16^a^, *rpl*20, *rpl*22, *rpl*23, *rpl*32, *rpl*33, *rpl*36
	Ribosomal RNAs	*rrn*4.5^1^, *rrn*5^1^, *rrn*16^1^, *rrn*23^1^
	Transfer RNAs	tRNA-Lys^a^,tRNA-Gln,tRNA-Ser,tRNA-Gly^a^,tRNA-Arg,tRNA-Cys,tRNA-Asp,tRNA-Tyr,tRNA-Glu, tRNA-Thr,tRNA-Ser,tRNA-Gly,tRNA-Met,tRNA-Ser,tRNA-Thr,tRNA-Leu,tRNA-Phe,tRNA-Val, tRNA-Gly,tRNA-Met,tRNA-Trp,tRNA-Pro,tRNA-Ile,tRNA-Leu^a^,tRNA-Val^a^,tRNA-His, tRNA-Ile^a^^1^, tRNA-Ala^a1^,tRNA-Arg^1^,tRNA-Asn^1^,tRNA-Leu,tRNA-Asn,tRNA-Arg,tRNA-Ala,tRNA-Ile,tRNA-His
	DNA-dependent RNA polymerase	*rpo*A, *rpo*B, *rpo*C1^a^, *rpo*C2
**Other genes**	Maturase	matK
	Protease	clpP^b^
	Envelope membrane protein	cemA
	Subunit acetyl-CoA-carboxylase	accd
	c-Type cytochrome synthesis gene	ccsA
**Genes of unkown function**	Conserved open reading frames	ycf1, 2a, 3^b^, 4, 15

^a^represents a gene with one intron

^b^represents a gene with two introns

^c^represents trans-splice gene

### SSR and codon usage analysis

**Fig. 2:**
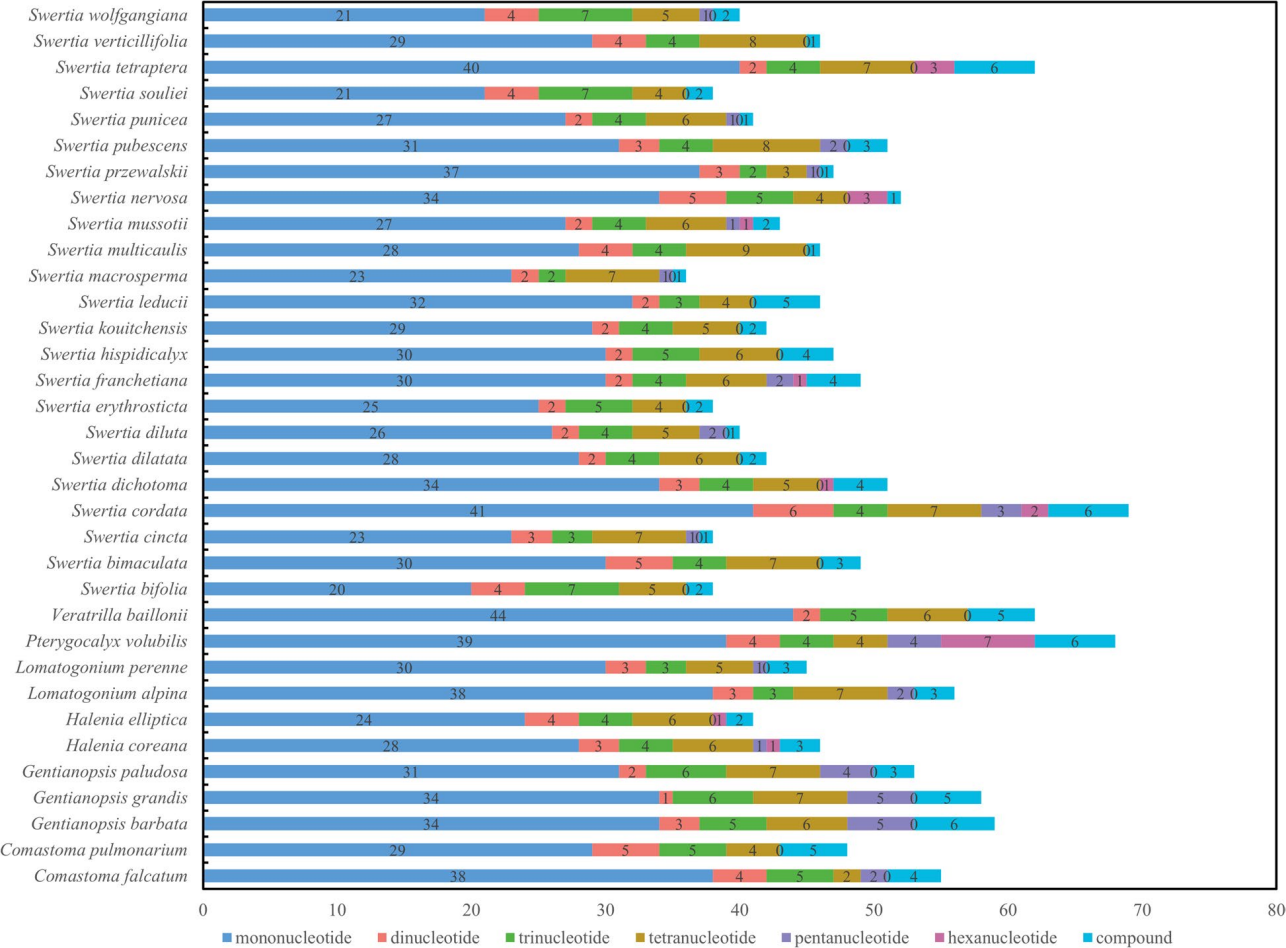
Simple sequence repeats (SSRs) in the 34 Subtribe Swertiinae plastid genomes

**Fig. 3:**
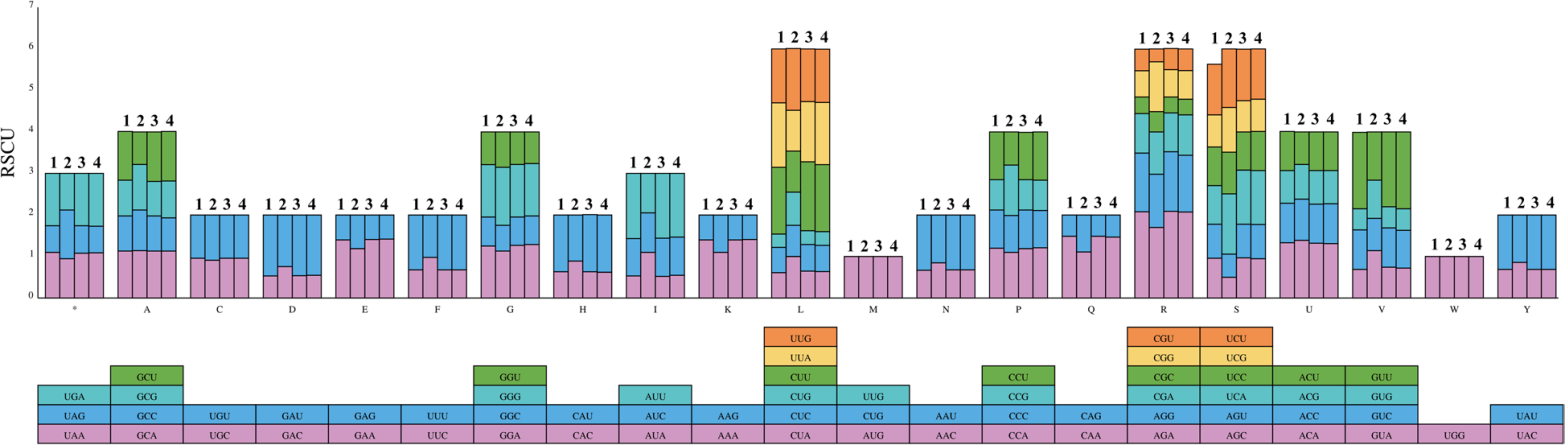
Codon contents of 20 amino acids and stop codons in all protein-coding genes of four Subtribe Swertiinae species chloroplast genomes. The top panel shows the RSCU for the corresponding amino acids. The colored block represents different codons, which are shown in the panel below. (Note: 1 represents *Halenia elliptica*; 2 represents *Swertia tetraptera*; 3 represents *S. mussotii*; 4 represents *Veratrilla baillonii*; A represents Alanine; C represents Cysteine; D represents Aspartic acid; E represents Glutamic acid; F represents Phenylalanine; G represents Glycine; H represents Histidine; I represents Isoleucine; K represents Lysine; L represents Leucine; M represents Methionine; N represents Asparagine; P represents Proline; Q represents Glutamine; R represents Arginine; S represents Serine; U represents Threonine; V represents Valine; W represents Tryptophan; Y represents Tyrosine)

The number of SSRs identified in the 34 subtribe Swertiinae chloroplast genomes ranged from 36 (*S. bifolia* Batalin and *S. erythrosticta* Maxim.) to 63 (*S. cordata* Wall.) (Fig. 2). Six types of repeat patterns were found among the SSRs, the numbers and types of which were different among the 34 chloroplast genomes. Among the mononucleotide repeats, A/T was dominant (50–82.22%), while C/G was rare (0–10.53%). Dinucleotides (1.89–11.63%), trinucleotides (4.35–19.44%) and pentanucleotides (3.92–20.00%) were found in all samples. Tetranucleotides and hexanucleotides were identified in eighteen and nine samples, respectively (Fig. 3 and Table S3).

Codon usage frequency of the 34 subtribe Swertiinae chloroplast genomes was detected based on the sequences of protein-coding genes (CDSs). The number of codons of protein-coding genes in the 34 chloroplast genomes ranged from 20,531 (*S. tetraptera*) to 26,402 (*H. elliptica*). In all species, serine (Ser; 1075–2268 instances) was the most abundant amino acid encoded by four codons, followed by arginine (Arg; 1137–2244 instances), encoded by six codons (Table S4). In contrast, methionine and tryptophan were encoded by only one codon, with instances ranging from 219 to 610 and from 387–605, respectively, and showed no codon usage bias (RSCU = 1). The AGA codon for arginine had the largest RSCU values (1.70–2.11), and the CUG codon for leucine had the smallest RSCU values (0.31–0.80) in the 34 chloroplast genomes. A total of 26 codons with RSCU values greater than one were identified among the 64 codons in the chloroplast genomes. Twenty-three of the 26 codons with RSCU values greater than one ended with A or U, which showed the codon preferences in the chloroplast genomes (Fig. 3, Table S4).

GC content of 34 CDS sequences ranged from 0.384 to 0.402, while GC3s content of 34 CDS sequences was between 0.282 and 0.451(Table 3). The average content of T3s, C3s, A3s, G3s, GC3s and GC content of 34 CDS sequences were 0.4122, 0.2518, 0.3765, 0.2368, 0.372 and 0.397 respectively (Table 3). The average ENC value was 53.64, the minimum ENC value of *S. franchetiana* was 50.62, and the highest value of *S. tetraptera* was 57.76 (Table 3). All ENC values of this subtribe were greater than 49, reflecting weak codon bias overall. The CAI value of all species ranged from 0.149–0.164.

**Table 3:** Codon usage of the Subtribe Swertiinae species. *T3s/C3s/A3s/G3s/GC3s* represents the thymine/cytosine/adenine/guanine/ GC content at synonymous third codon position, *GC* notes the total GC content, *CAI* represents codon adaptation index, *ENc* represents effective number of codons

Species	T3s	C3s	A3s	G3s	CAI	ENc	GC3s	GC
*Comastoma falcatum*	0.4124	0.2578	0.3724	0.238	0.163	53.67	0.377	0.397
*Comastoma pulmonarium*	0.4122	0.2586	0.3700	0.2395	0.163	53.77	0.379	0.398
*Gentianopsis barbata*	0.4104	0.2581	0.3742	0.2384	0.162	53.79	0.377	0.396
*Gentianopsis grandis*	0.4581	0.1773	0.4242	0.1892	0.163	50.70	0.281	0.384
*Gentianopsis paludosa*	0.4113	0.2578	0.3740	0.2383	0.162	53.73	0.377	0.396
*Lomatogoniopsis alpina*	0.4124	0.2579	0.3728	0.2391	0.164	53.70	0.377	0.396
*Lomatogonium perenne*	0.4123	0.2577	0.3728	0.2389	0.164	53.70	0.377	0.396
*Pterygocalyx volubilis*	0.4128	0.2573	0.3732	0.237	0.163	53.76	0.376	0.396
*Veratrilla baillonii*	0.4081	0.2583	0.3751	0.2382	0.163	53.90	0.378	0.399
*Halenia coreana*	0.4107	0.26	0.3634	0.2402	0.161	53.94	0.382	0.402
*Halenia elliptica*	0.4109	0.2582	0.3727	0.237	0.162	53.73	0.377	0.397
*Swertia bifolia*	0.4107	0.2595	0.3734	0.2368	0.164	53.79	0.377	0.397
*Swertia bimaculata*	0.4114	0.2585	0.3735	0.2373	0.163	53.71	0.377	0.397
*Swertia cincta*	0.4113	0.259	0.3721	0.2391	0.163	53.76	0.378	0.397
*Swertia cordata*	0.4117	0.2586	0.3728	0.2367	0.163	53.78	0.377	0.397
*Swertia dichotoma*	0.4108	0.2584	0.3738	0.2371	0.162	53.80	0.377	0.397
*Swertia dilatata*	0.4115	0.2586	0.3722	0.2386	0.163	53.78	0.378	0.397
*Swertia diluta*	0.4097	0.2605	0.3633	0.2418	0.162	54.00	0.383	0.403
*Swertia erythrosticta*	0.4110	0.2589	0.3737	0.2368	0.163	53.81	0.377	0.396
*Swertia franchetiana*	0.4557	0.1778	0.4231	0.1884	0.162	50.62	0.282	0.387
*Swertia hispidicalyx*	0.4115	0.2591	0.372	0.2382	0.163	53.77	0.378	0.397
*Swertia kouitchensis*	0.4107	0.259	0.372	0.2382	0.163	53.85	0.378	0.398
*Swertia leducii*	0.4109	0.2588	0.3732	0.2373	0.163	53.84	0.377	0.397
*Swertia macrosperma*	0.4103	0.2603	0.3714	0.2372	0.163	53.82	0.379	0.398
*Swertia multicaulis*	0.4109	0.2579	0.3724	0.2391	0.162	53.81	0.378	0.397
*Swertia mussotii*	0.4111	0.2597	0.3711	0.2373	0.163	53.85	0.378	0.398
*Swertia nervosa*	0.4101	0.2592	0.3728	0.2381	0.163	53.78	0.378	0.397
*Swertia przewalskii*	0.4107	0.2591	0.3733	0.2373	0.163	53.78	0.377	0.397
*Swertia pubescens*	0.4118	0.2596	0.3716	0.2391	0.164	53.77	0.378	0.397
*Swertia punicea*	0.4108	0.2589	0.3722	0.2382	0.164	53.86	0.378	0.398
*Swertia souliei*	0.4097	0.2605	0.3664	0.2396	0.162	53.89	0.381	0.401
*Swertia tetraptera*	0.3115	0.2644	0.3721	0.3384	0.149	57.76	0.451	0.410
*Swertia verticillifolia*	0.4570	0.1781	0.4229	0.1894	0.162	50.77	0.282	0.385
*Swertia wolfgangiana*	0.4111	0.2588	0.3736	0.2369	0.163	53.79	0.377	0.396
Mean	0.4122	0.2518	0.3765	0.2368	0.162	53.64	0.372	0.397

### Comparative genomice analysis

**Fig. 4:**
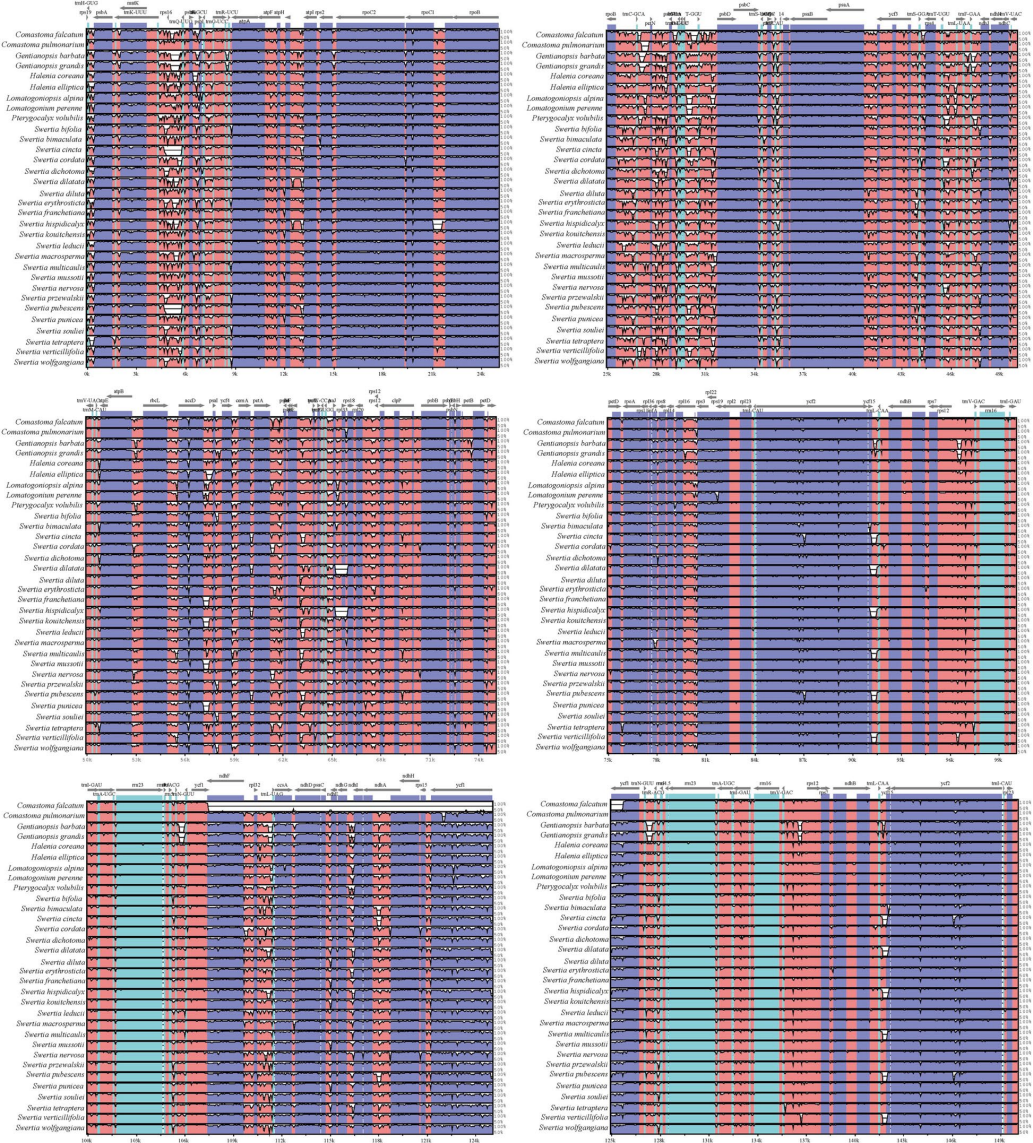
Comparison and analysis based on chloroplast genome of 34 Subtribe Swertiinae species. Orientation of genes were pointed out by arrows up the alignments. Purple, blue, pink and grey bars correspond to exons, untranslated regions, non-coding sequences and mRNA respectively. Y-axis indicates the genetic similarity percentage. Genetic similarity among 50-100% were showed in the figure. (For interpretation of the references to colour in this figure legend, the reader is referred to the web version of this article.)

**Fig. 5:**
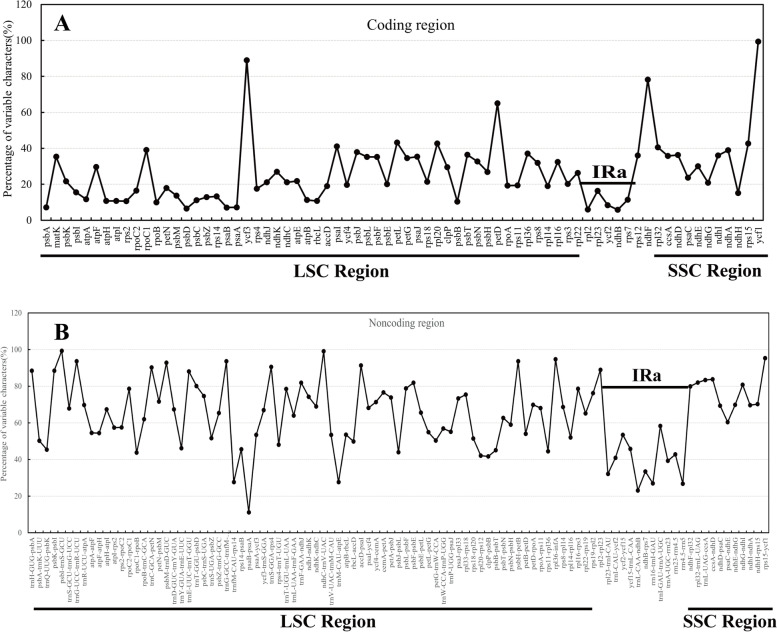
Percentages of variable characters in homologous regions among chloroplast genomes of 34 Subtribe Swertiinae species. **A** Coding region. **B** Noncoding region. The homologous regions are oriented according to their locations in the chloroplast genome

We used the online tool mVISTA to identify the potential divergent sequences among the 34 subtribe Swertiinae chloroplast genomes, with the chloroplast genome of *V. baillonii* as a reference. The structures and sequences of the chloroplast genomes were conserved, especially in the IR regions (Fig. 4). Meanwhile, we used DNASP software to calculate the variation rate of coding and noncoding regions. The variation rates of noncoding regions were generally higher than those of coding regions (Fig. 5). The variation in noncoding region genes ranged from 11.11 to 99.28%, with an average of 63.98%, whereas the variation in coding region genes ranged from 5.78 to 88.97%, with an average of 25.39%. The variation rates of both coding regions and noncoding regions in the IR region were lower than those in other regions. Additionally, the noncoding intergenic regions were highly divergent, especially *trnC-GCA-petN*, *trnS-GCU-trnR-UCU*, *ndhC-trnV-UAC*, *trnC-GCA-petN*, *psbM-trnD-GUC*, *trnG-GCC-trnfM-CAU*, *trnS-GGA-rps4*, *ndhC-trnV-UAC*, *accD-psaI*, *psbH-petB*, *rpl36-infA* and *rps15-ycf1*. However, highly divergent regions were also found within protein-coding regions, such as in *ycf3*, *petD*, *ndhF*, *petL*, *rpl20*, *rpl15* and *ycf1*. In addition, no genomic rearrangements were detected in the alignment analysis of 34 subtribe Swertiinae chloroplast genomes.

### Gene selective pressure analysis

**Fig. 6:**
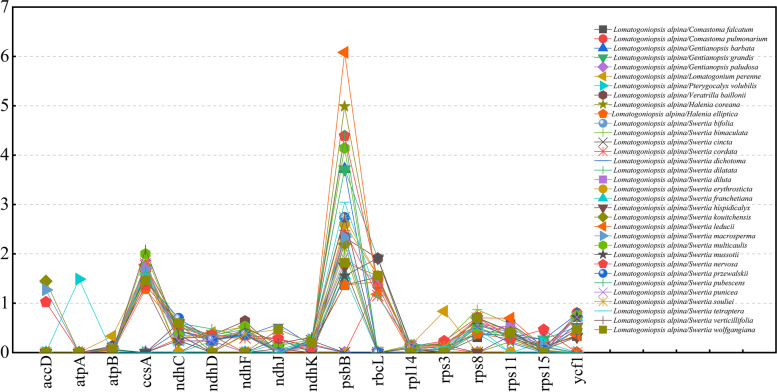
The *Ka*/*Ks* ratio of 80 protein-coding genes of 33 chloroplast genomes for comparison with *Lomatogoniopsis alpina*. *Ka*/*Ks* ratio > 1 indicates strong positive selection

We calculated the nonsynonymous (Ka) and synonymous (Ks) substitution ratios for 80 protein-coding genes to estimate the selection pressure on chloroplast genes by comparing *L. alpina* T.N.Ho & S.W.Liu with 33 other species in subtribe Swertiinae. The ratios of sixty-three protein coding genes could not be calculated because of Ka or Ks = 0, demonstrating that no synonymous or nonsynonymous changes occurred. For the remaining 17 protein-coding genes, the results indicated that the mean Ka/Ks ratio between *L. alpina* and the 33 other subtribe Swertiinae species ranged from 0.01 (rpl14) to 2.34 (*psb*B) (Fig. 6). However, the Ka/Ks ratio for most genes was less than one, showing that they underwent negative selection, except for *ccs*A and *psb*B, which experienced positive selection (Ka/Ks > 1).

### Phylogenetic analysis

**Fig. 7:**
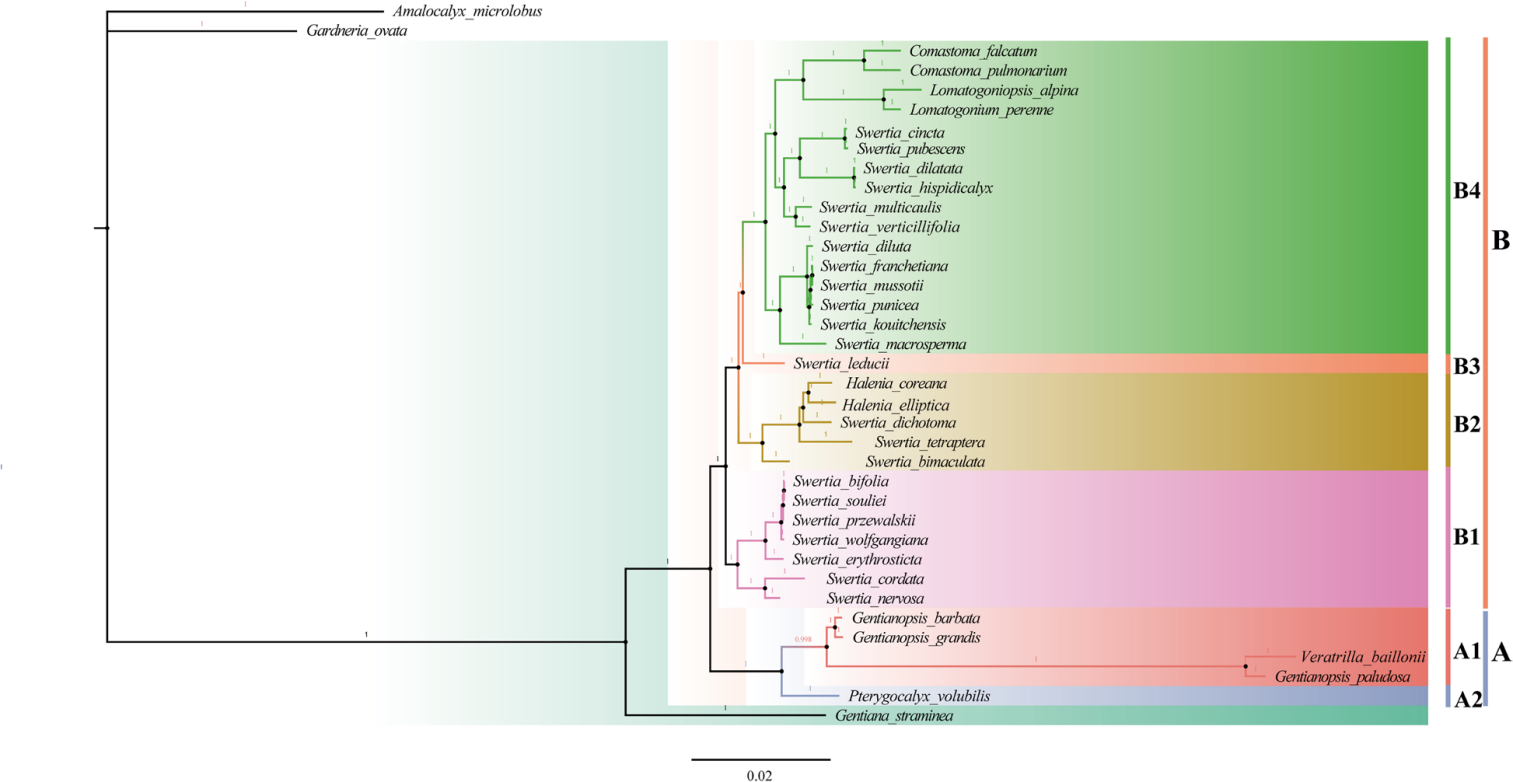
Phylogenetic tree of 34 Subtribe Swertiinae species using Bayesian inference (BI) analyses based on whole chloroplast genomes. Numbers at nodes correspond to Bayesian inference (BI) posterior probabilities (only probabilities ≥ 0.95 are shown). Different colors represent different clades: bule represents A1, red represents A2, purple represents B1, chartreuse represents B2, pink represents B3 and green represents B4

We used the complete chloroplast genome sequences and 80 shared protein sequences of 34 species from subtribe Swertiinae to construct phylogenetic trees with *G. straminea*, *G. ovata* and *A. microlobus* as outgroups. Phylogenetic trees built with the whole chloroplast genome and CDSs had the same topology (Figure S1). The Bayesian trees demonstrated that all species in the subtribe Swertiinae formed a monophyletic clade with high support in term of Bayesian posterior probabilities (PP = 1; Fig. 7). Additionally, this well-supported clade was divided into two major clades (A and B) within subtribe Swertiinae. Clade A was located at the base of the phylogenetic tree and was divided into two subclades (A1 and A2). The A1 subclade (*P. volubilis*) was sister to the A2 subclade consisting of three species of *Gentianopsis* and *V. baillonii*. Interestingly, *G. paludosa* did not cluster with the other two species of the same genus but clustered with *V. baillonii,* indicating that *G. paludosa* was closely related to *V. baillonii*. Clade B contained 29 species from the remaining 6 genera of subtribe Swertiinae, which formed three main branches in the phylogenetic tree (B1, B2 and B4), that is, the subgen*. Swertia* branch (B1), Gen. *Halenia- Swertia dichotoma*- Gen. *Sinoswertia*- *Swertia bimaculate* branch (B2) and subgen. *Ophelia*-Gen. *Comastoma*-*L. alpina*-*Lomatogonium perenne* T.N. Ho & S.W. Liu ex J. X. Yang branch (B4).

### Divergence time estimate

**Fig. 8:**
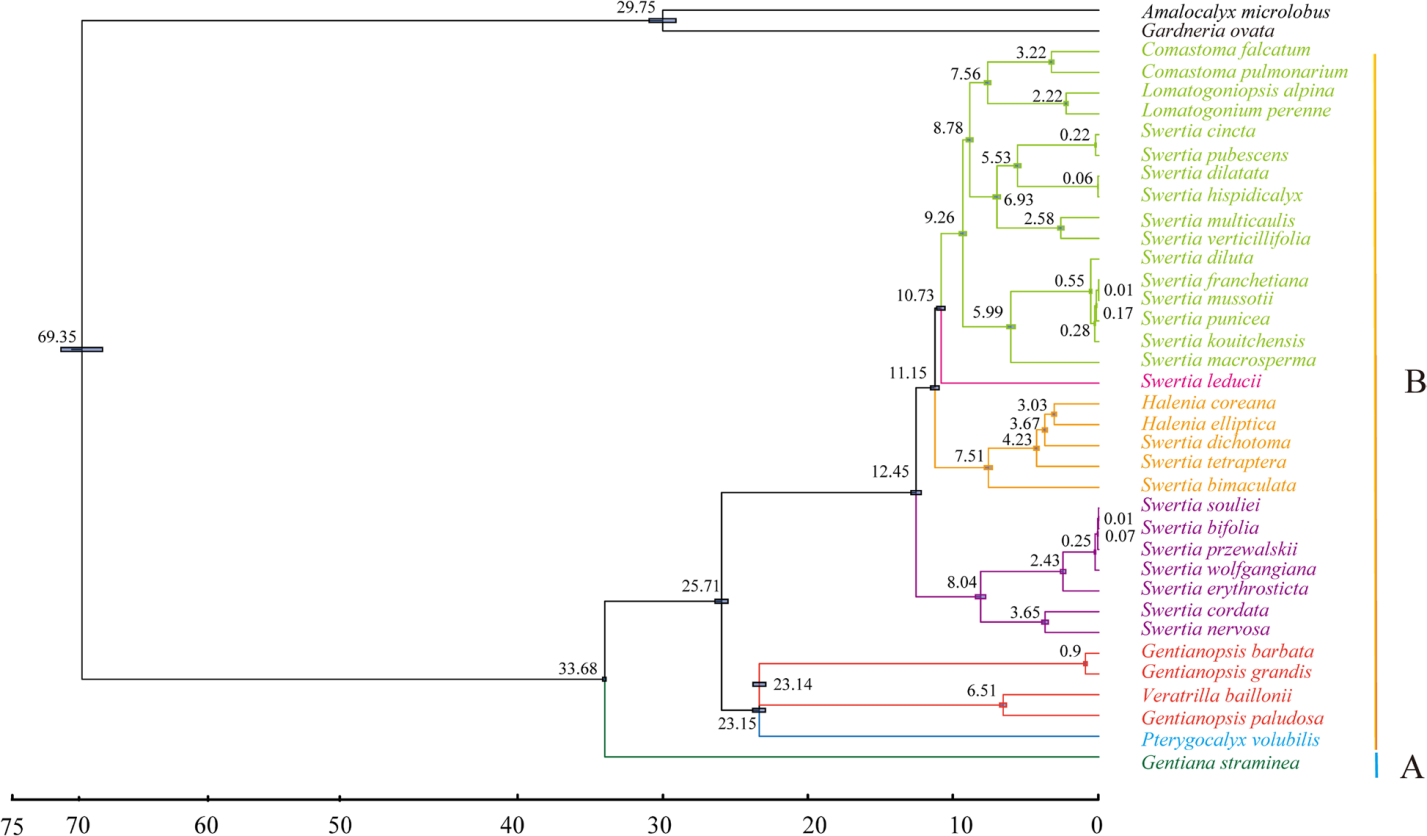
Divergence times of 34 species in Subtribe Swertiinae

In this study, complete chloroplast genomes sequences of 34 subtribe Swertiinae plants and three outgroups were used to estimate the divergence times of major clades in the subtribe Swertiinae. The divergence between subtrib Gentianinae and subtrib Swertiinae was estimated to occur in 33.68 Ma (Fig. 8). Roate group and Tubular group in Subtribe Swertiinae approximately diverged in 25.17 Ma. As far as the current 9 genera were concerned, *Pterygocalyx* was the oldest group of subtribe Swertiinae, diverged at 24.13 Ma. And then, *Gentianopsis* and *Veratrilla* diverged at 24.13 Ma. *Swertia* probably originated at 12.45 Ma. *Comatoma*, *Lomatogoniopsis* and *Lomatogonium* constituted the most evolved clades of subtribe Swertiinae, diverged probably at 7.56 Ma. The estimated divergence time in 23 species of *Swertia* was between 7.51 and 0.01 Ma.

### IR contraction and expansion

**Fig. 9:**
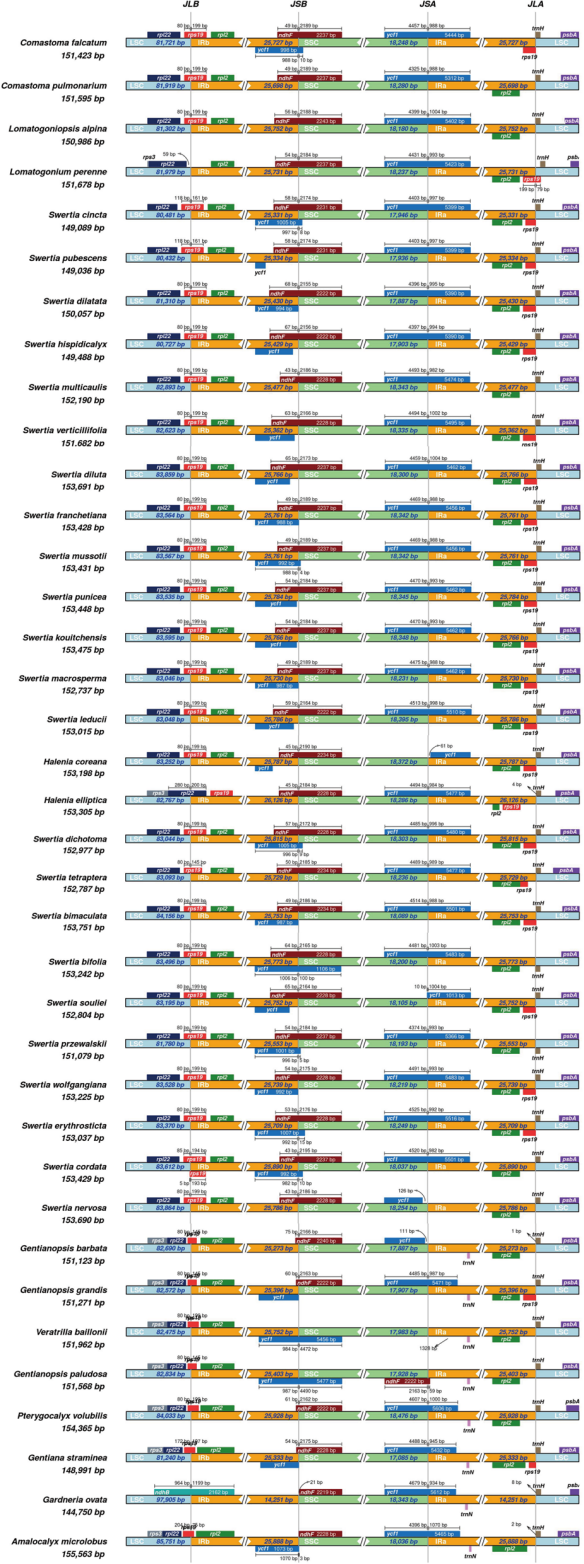
Contraction and expansion of inverted repeats at the junction of chloroplast genome. JLB: LSC/IRb; JSB: IRb/SSC; JSA: SSC/IRa; JLA: IRa/LSC. Arrows illustrate the distance of genes from the junction site as shown for *rpl*22 at JLB and for *trn*H at JLA. The scale bar above some genes illustrates the number of base pairs that each gene occupies in specific regions of the chloroplast, e.g., the scale bar above *ndhF* represents the part of the gene located in the IRb region and the SSC region

We used the IRscope online website (https://irscope.shinyapps.io/irapp/) to visualize the differences in the four boundaries of the LSC, SSC, and IRs. Comparison of all subtribe Swertiinae plastomes with three outgroups uncovered relatively stable IRs, with little expansion or contraction (Fig. 9). In these 37 plastomes, the LSC-IRa borders were located in the *rps19* gene with the exception of the LSC-IRa border of *L. perenne*, *H. elliptica* and *G. ovata*. In the outgroup *G. ovata*, the LSC-IRa border was located within the *ndhB* gene, while in *L. perenne,* the LSC-IRa border was located in the *rpl22* gene, showing that the border had shifted by 59 bp. In *H. elliptica*, the LSC-IRa border was located within the *rpl22* gene, which had undergone contraction. The boundary of SSC-IRa was positioned in the *ndhF* gene, *ycf1* pseudogene and the intergenic spacer region between the *ycf1* pseudogene and *ndhF*. The exact position of the SSC-IRb border had shifted by 10 bp in *C. falcatum*, 8 bp in *S. cincta* Burkill, 4 bp in *S. mussotii*, 9 bp in *S. dichotoma* L., Sp. Pl., 5 bp in *S. przewalskii* Pissjauk*.*, 15 bp in *S. erythrosticta*, 10 bp in *S. cordata* and 3 bp in the outgroup *A. microlobus*. The SSC/IRa border in all subtribe Swertiinae plastomes was located within the *ycf1* gene with a few exceptions, and the sequences demonstrated length variabilities among species. The IRa/LSC border in most species’ chloroplast genomes was located at the junction of the *trnH* gene and the *rps19* pseudogene. In the *L. perenne* chloroplast genome, the *trnH* gene was far inside the LSC region, and *the rps19* pseudogene was positioned at the IRa/LSC border. In *V. baillonii*, *L. alpina*, *G. paludosa*, *Gp. Barbata* (Froel.) Ma,* C. pulmonarium*,* S. przewalskii*, *S. nervosa* Wall., *S. multicaulis* D.Don and *S. cordata* chloroplast genomes, *rps19* pseudogenes were lost, and the IRa/LSC border was positioned at the *trnH* gene.

## Discussion

### Organization and features of chloroplast genomes

Our study compared the features, content, and organization of the chloroplast genomes of 34 species in subtribe Swertiinae, demonstrating that all of them exhibited the typical quadripartite structure found in vascular plants [34–36]. The length of the chloroplast genomes of the 34 species varied from 149,036 (*S. pubescens*) to 154,365 bp (*P. volubilis*), implying that they are relatively conserved, revealing only minor differences that changed their sizes. Differences in chloroplast genome length within genera and families have previously been reported, such as in *Swertia* (Gentianaceae) [27], *Notopterygium* (Apiaceae) [37] and *Rhodiola* (Crassulaceae) [38], and in the subfamily Coryloideae of Betulaceae [39]. In this study, the differences in the chloroplast genome length of 34 species in 9 genera of subtribe Swertiinae were mainly caused by expansion and contraction of the IR region [40].

The chloroplast genomes had similar GC contents (37.5-38.26%), indicating high species similarity. The GC content in the IR region (43.39%) was higher than that in the other two regions (LSC, 35.92%; SSC, 31.88%), which may be related to the presence of four rRNA sequences in these regions, e.g., rrn16, rrn23, rrn4.5, and rrn5, as previously reported in many complete chloroplast genomes of angiosperms [41].

Regarding gene estimates, we found some differences among the chloroplast genomes. Gene numbers ranged from 129 (*Gp. paludosa*) to 134 (*Gp. grandis*, *V. baillonii*, *H. coreana*, *S. bimaculate*, *S. diluta*, *S. leducii*, *S. mussotii*, *S. souliei*, *S. tetraptera*, *S. verticillifolia* and *S. wolfgangiana*). *Gp. paludosa* had 129 genes due to the absence of the *ycf15* gene and pseudogenes *rps16*, *rps19*, i*nfA* and *ycf1*, while *Gp. grandis*, *H. coreana*, *S. bimaculate*, *S. diluta*, *S. leducii*, *S. mussotii*, *S. souliei*, *S. tetraptera*, *S. verticillifolia* and *S. wolfgangiana* contained 134 genes because of a duplication of *rps19* and *ycf1*. In fact, duplicated *rps19* and *ycf1* pseudogenes have also been reported in other Gentiaceae species [42]. Similarly, *rpl33* was lost from *S. dilatate*, *S. hispidicalyx*, *P. volubilis* and *C. pulmonarium*. According to relevant research on *rpl33*, this gene could maintain enough plastid translation ability in cold environments [43]. In the light of our results and previous studies, *rpl33* exists in most of Gentianaceae members with wide distribution and large numbers of species. In contrast, rpl33 is not as common in less specious, like *Gentianella* and *Pterygocalyx*. Therefore, the gene *rpl33* may confer greater tolerance of cold climates; this explains the relatively smaller number of *Gentianella* and *Pterygocalyx* on QTP. Additionally, there have been reports of the absence of *ndh* genes in other Gentiaceae species, including *ndhA*, *ndhC*, *ndhG*, *ndhH*, *ndhI*, *ndhJ*, and *ndhK* [44]. However, the lack of the *ycf15* gene has not been reported. Thus, small changes in the content of these genes in the chloroplast genomes of subtribe Swertiinae are caused by evolutionary events of gene deletion and insertion.

### Simple sequence repeats (SSRs) and codon usage analysis

Chloroplast SSRs usually show a high level of variation and are widely used in the study of polymorphism, population genetics and phylogenetics [29, 45, 46]. Our study analyzed the number of different SSR motifs in the chloroplast genomes of 34 subtribe Swertiinae species. Compared with that in other angiosperms, the number of chloroplast genome SSRs (36–63) in the 34 subtribe Swertiinae species was low to medium. Among the SSRs, a large number of single nucleotide repeats were detected, among which polyA and polyT structures were common, which was consistent with the results of previous studies [47–50]. These SSRs may be useful for subsequent analyses of interspecies genomic polymorphism and population genetics based on repeat length polymorphism. Experts have different views of the mechanisms explaining most SSRs in chloroplast genomes. Slip chain mismatch and intramolecular recombination are currently considered the main mechanisms that cause most SSRs [51].

The choice of synonymous codons for amino acids encoded by an organism’s genes is not completely random, and there is codon usage bias. Codon usage bias not only plays an important regulatory role in gene expression level but also helps to improve the accuracy and efficiency of translation. In addition to being affected by selection and mutation, codon usage is affected by tRNA abundance, base composition, gene position on chromosomes, gene length and expression level, amino acid hydrophobicity and mRNA secondary structure.

Previous studies have shown that analysis of codon bias in the chloroplast genome is helpful for understanding the origin and evolution of species [52]. In addition, the frequency of codon use is also related to gene expression. Nucleotide composition is one of the important factors affecting codon use bias. In the genome, the AT and GC contents are closely related to synonymous codon use bias. GC refers to the total content of all codons G and C, and GC3s pertains to the frequency of G and C bases in the third codon base of synonymous codons encoding the same amino acid. GC reflects the strength of directional mutation pressure, and GC3s is closely related to codon bias. The main difference in synonymous codons is reflected in the third base, and a change in this base of codons usually does not cause changes in encoded amino acids. Therefore, the selection pressure on the third base of the codon is less selective [53]. GC3s is used as an important basis for analyzing codon usage pattern [54]. GC and GC3s in the codons of these 34 chloroplast genomes were all less than 0.5, indicating that the chloroplast genomes of Subtribe Swertiinae species tended to use A/T bases and A/T-ending codons. Moreover, most amino acids in the 34 species chloroplast genomes had codon bias with a high preference (RSCU > 1), except for methionine and tryptophan (RSCU = 1). The RSCU value of codon types ending with A or U was larger than that for types ending with G or C, which showed a codon preference for bases A or U in the 34 species’ chloroplast genomes. Similar conclusions have been made in studies of Phyllanthaceae [55], *Notopterygium* [37], *Cinnamomum camphora* [56] and others. Codon adaptation index values and effective number of codon values indicated a slight bias in codon usage in the Subtribe Swertiinae species. CAI and ENC are important indexes to judge strength of codon bias [57]. In this study, CAI ranged from 0.149 to 0.164 in chloroplast genomes of 34 Subtribe Swertiinae species, with an average of 0.162, while ENc ranged from 50.62 to 57.76 in chloroplast genomes of 34 Subtribe Swertiinae species, with an average of 53.64. These results indicated that codon bias was weak in chloroplast genomes of Subtribe Swertiinae species, which might be related to the conserved of chloroplast genomes of Subtribe Swertiinae species [57]. In all, these findings advance our understanding of the evolutionary history of subtribe Swertiinae, especially in relation to natural selection and mutation pressures.

### Comparative genomes and characterization of substitution rates

Although the chloroplast genome is considered to be fairly conserved in angiosperms, mutational hotspots are often found in the sequences of some closely related species. These mutational hotspots are widely used in plant phylogenetics, group genetics and DNA barcode research. Based on the nucleotide diversity analyses, we identified nineteen highly variable regions with high variation rates that can be used as markers to resolve taxonomic issues in subtribe Swertiinae and function as DNA barcodes for species identification.

The intergenic regions *ndhC-trnV-UAC*, *trnC-GCA-petN*, *trnS-GCU-trnR-UCU*, *ndhC-trnV-UAC*, *trnC-GCA-petN*, *psbM-trnD-GUC*, *trnG-GCC-trnfM-CAU*, *trnS-GGA-rps4*, and *ndhC-trnV-UAC* possess high variation rates (Fig. 5), however, these markers are rarely used in plant phylogenetics. The intergenic region *accD-psaI* is approximately 640 bp long and is used for research in *Gentiana sect. Cruciata* (Gentianaceae) [58], Pyrus (Rosaceae) [59], and Coryloideae (Betulaceae) [60]. Compared with existing candidate genes (*matK* and *rbcL* combination), the *ycf1* gene is more suitable for barcodes of land plants due to its more variable loci [61]. Recently, *ycf1* gene has been treated as the core DNA barcode in sect. *Cruciata* and found to provide sufficient genetic markers for resolving the phylogeny of Gentianaceae [58]. Based on our study, these two divergent markers may be useful for further phylogenetics and species identification of subtribe Swertiinae.

### Phylogenetic and divergence time of Subtribe Swertiinae

The topologies of the ML and BI trees constructed with complete chloroplast genome sequences and shared protein-coding gene sequences were consistent, indicating that all 34 subtribe Swertiinae species formed a monophyletic clade, which was sister to Subtribe Gentianaceae. The monophyly of subtribe Swertiinae is therefore verified by chloroplast genome data, a finding consistent with that of previous studies [4–8]. Meanwhile, our molecular phylogeny agreed with the taxonomic classification of subtribe Swertiinae in the Roate group and Tubular group. Previous studies have shown that the Roate group contains *Swertia*, *Lomatogonium*, *Lomatogoniopsis* and *Sinoswertia*, while the Tubular group includes the rest. In this study, the well-supported clade was divided into two major clades (A and B) within subtribe Swertiinae, of which clade B represented the Roate group (except for *Halenia*) and clade A represented the Tubular group. *P. volubilis* was closely related to *Gentianopsis and V. baillonii*, which are located at the base of subtribe Swertiinae. In the other studies, the base groups of subtribe Swertiinae also included *Obliaria*, *Latouchea*, *Bartonia* and *Megacodon*. From the analysis of geographical distribution, the basal groups are mostly isolated monospecies or small genera containing only a few species, such as *Obliaria* (1 species) and *Bartonia* (4 species), distributed in North America. *Latouchea* (1 species) and *Megacodon* (2 species) are distributed in Southwest China and the Himalaya region. *Pterygocalyx* (1 species) is distributed in Asia, and *Veratrilla* (2 species) is distributed in southwest China, northeast India, Sikkim and Bhutan. From the perspective of morphology, except for *Bartonia* (no floral nectary was observed), *Obliaria*, *Megacodon*, *Latouchea*, *Gentianopsis* and *Pterygocalyx* all have floral nectaries at the base of the ovary, which is the same as *Gentian* of the outgroup and different from other genera of subtribe Swertiinae (most species have floral nectaries on corolla lobes). Thus, nectaries at the base of the ovary may be ancestral characteristics of subtribe Swertiinae. However, the position of *V. baillonii* is still unresolved because the results of this study were inconsistent with those of previous studies [25, 26, 28]*. C. falcatum* and *C. pulmonstium* formed a clade, which showed that *Comastoma* was as one monophyletic group. Our result supports previous studies based on ITS sequences and embryological characteristics [12, 62]. *Comastoma* clustered with *L. perenne* and *L. alpina* in a more recently evolved branch. The genus *Halenia* is closely related to *S. tetraptera*, *S. dichotoma* and *S. bimaculata*, which is consistent with the results of molecular systematics study based on ITS fragments by von Hagen and Kadereit (2002) [9] and embryology study by Xue et al. (2007) [63]. In addition, *Halenia*, *Lomatogonium*, *Lomatogoniopsis*, and *Sinoswertia* were polyphyletic with the closely related ally *Swertia*, whereas V*eratrilla* was polyphyletic with its closely related ally *Gentianopsis*, which indicated that these genera were not monophyletic groups. The molecular data of these genera conflicted with their morphological classification, which was consistent with the findings of previous studies. The inconsistencies may be explained by the fact that the morphological classification does not reflect the true evolutionary history. The most significant reason is the plasticity of morphological characteristics.

Exploitation and habitat destruction have seriously threatened the resources of famous Tibetan folk medicinal species. Phylogenetic relationships have substantial scientific significance for the sustainable use of rare resources of alpine medicinal plants. For example, the resources available for *S. mussotii,* the original traditional Tibetan folk medicinal species called “Dida”, cannot not meet the increasing market demand. An in-depth study on the genetic relationships of medicinal plants in subtribe Swertiinae showed that *S. franchetiana*, *S. punicea* and *S. kouitchensis* were closely related to *S. mussotii*, which has guiding significance for expanding drug sources.

Phylogenetic and divergence time analysis indicated that the subtribe Swertiinae species may have undergone rapid radiation. The divergence time analysis results indicated that subtribe Swertiinae originated in the end of the Eocene (Fig. 9). Two significant periods of rapid diversification of subtribe Swertiinae were found. The first was in the early Miocene. During this period, the QTP was further uplifted, and the Himalayan mountains and Tianshan Mountains were significantly elevated, which strongly changed the atmospheric circulation. Meanwhile, the global temperature decreased from the optimum temperature in the middle Miocene of the third century, resulting in a cool and dry climate [64–67]. The cool and dry climate resulted in suitable habitats for subtribe Swertiinae fragmentation, which led to the first rapid radiation and.led to the major lineages of the extant subtribe Swertiinae species. The second period was in the Quaternary, which led to most of the extant subtribe Swertiinae species. After 5 Ma, the global temperature decreased sharply after a short period of global warming [68], providing a diverse range of habitats and further increasing the species diversity of subtribe Swertiinae*.*

### Adaptative evolution of subtribe Swertiinae

Synonymous and nonsynonymous nucleotide substitution patterns play a major role in adaptive evolution. In subtribe Swertiinae, we did not detect significant positive selection for the majority of genes, with only two genes (*ccsA* and *psbB*) showing possible positive selection; these may have played a vital role in adaptive evolution in subtribe Swertiinae. Our results were in accordance with those of a previous study, which showed that *ccsA* was under positive selection in the chloroplast genome of 15 angiosperms [69]. *psbB*, encoding photosystem subunits (Table 2), plays a vital role in the life history of plants. In addition, the *ccsA* gene is a c-type cytochrome synthesis gene (Table 2) in plants. *The cssA* gene is responsible for encoding the cytochrome c synthesis protein, which has approximately 250 ~ 350 amino acids and is a membrane binding protein. The coding product of ccsA can co-form the *ccsA* complex with the coding protein of another gene, *ccsB* [70]. Xie et al. (1996) [71] believed that the *ccsA* gene was related to the binding of cytochrome C-heme. This has implications for understanding the adaptive evolution of *ccsA* genes in angiosperms. These genes are highly correlated with physiological processes such as photosynthesis; thus, their positive selection on them may help subtribe Swertiinae species quickly adapt to all kinds of environments and enable their wide global distribution.

Plant species in subtribe Swertiinae are diverse and have important medicinal value. The current analysis of the chloroplast genome has revealed its unique sequence structure and rich genetic information. However, for the whole subtribe, the use of only 34 genome sequences is far from sufficient. In the next step, the genetic data of other taxa of this subtribe should be supplemented to further explore the phylogenetic relationships of this subtribe and provide a scientific basis for the conservation of species resources of this subtribe and the study of Chinese traditional medicine pharmacognosy.

Single-copy nuclear genes are helpful for elucidating the complex phylogenetic relationships of subtribe Swertiinae. Single-copy nuclear genes play an important role in molecular phylogenetics [72]. Compared with common nuclear genes, single-copy nuclear genes can reduce the influence of paralogous genes. Because of the large number of duplicate genes in the plant genome, it is difficult to screen single-copy nuclear genes [73]. However, with the development of high-throughput sequencing technology, it has been possible to develop a large number of single-copy nuclear genes at the whole-genome level [74, 75], and they have been applied in phylogenetic studies of several taxa [76]. However, no single-copy nuclear genes have been studied in subtribe Swertiinae. There are still many problems in the classification of subtribe Swertiinae, and the monophyly of several genera has not been determined, which reflects the complex evolutionary process of this Subtribe. Therefore, a large number of single-copy nuclear genes should be widely used to resolve the phylogenetic relationships of rapidly differentiated taxa such as those belonging to subtribe Swertiinae. Because the phylogenetic trees constructed from chloroplast data and nuclear gene data are often inconsistent [5], integrative analysis of the two is particularly important. At the same time, population genetics can be used to analyze the phylogenetic relationships and evolutionary processes of closely related taxa within genera or groups.

## Conclusion

We presented a comparative analysis of 34 plastomes from 34 subtribe Swertiinae species and reported a comprehensive study of their phylogenetic relationships, divergence time estimates, and adaptative evolution. Phylogenetic analysis showed that the 34 species formed a monophyletic clade, and *Veratrilla*, *Gentianopsis* and *Pterygocalyx* were located at the base of the phylogenetic tree. In addition, our molecular phylogenetic results agreed with the taxonomic classification of subtribe Swertiinae in the Roate group and Tubular group. Positive selection analyses showed that two genes (*ccsA* and *psbB*) had high Ka/Ks ratios, indicating that chloroplast genes may have undergone positive selection during the evolutionary history of this group. Divergence time analysis revealed that subtribe Swertiinae originated in the end of the Eocene and diversified in the early Miocene Tertiary. Overall, the whole chloroplast genome sequences provide valuable information for elucidating the phylogeny, divergence time and evolutionary process of subtribe Swertiinae.

## Materials and methods

### Sampling

We collected fresh young leaves of *S. tetraptera*, *S. franchetiana*, *S. przewalskii* and *S. bifolia*. from Mengyuan County of Qinghai Province (101.32′E, 37.62′N, 3,208 m), Huangzhong County of Qinghai Province (101.63′E, 36.57′N, 2,510 m), Qilian County of Qinghai Province (99.61′E, 38.83′N, 3,234 m), and Qilian County of Qinghai Province (102.22′E, 37.45′N, 3,135 m), respectively. We used silica gel to rapidly store the leaves until dried. These samples were all identified by taxonomist Prof. Yuhu Wu at the Northwest Institute of Plateau Biology, Chinese Academy of Sciences. Voucher specimens of these four species were deposited in the Qinghai-Tibetan Plateau Museum of Biology (QTPMB) with voucher numbers QHGC-2011, QHGC20190821, QHGC-2013, and QHGC-2014, respectively. All of the published complete chloroplast genomes of Subtribe Swertiinae were downloaded from GenBank (Table S5). In total, 34 plastid genomes from 9 genera of subtribe Swertiinae species were obtained. Our experimental research, including the collection of plant materials, complies with institutional, national, or international guidelines.

### DNA extraction, library preparation and genome sequencing

Total genomic DNA was extracted from dried leaves of the four *Swertia* L. plants by using an improved CTAB method [77] and estimated for purity and concentration using a NanoDrop 2000 microspectrophotometer. Each genomic DNA sample was broken into fragments of different lengths by ultrasound. Then, the DNA fragments were purified, the ends were repaired, an A tail was added to the 3' end, and the sequencing joints were connected. After that, agarose gel electrophoresis was used to select suitably sized DNA fragments, and PCR amplification was performed to complete the preparation of the sequencing library. After qualified library quality inspection, the Illumina HiSeq platform (Beijing Biomarker Technologies Co., Ltd.) was used for 150 bp paired-end sequencing.

### Chloroplast genome assembly and annotation

Raw sequencing data were transformed into sequenced reads (raw data) by performing a base calling analysis of the raw image files. SQCToolkit_v2.3.3 software [78] was used to filter the raw read data obtained by sequencing to remove low-quality regions and obtain clean reads. The results were then stored in the FASTQ format. We used the iterative organelle genome assembly pipeline to assemble the chloroplast genome with *S. mussotii* (NC_031155) serving as a reference [79]. Then, SPAdes v3.6.1 software was employed for de novo splicing under default parameters and to generate a series of contigs [80]. Contigs larger than 1,000 bp were used for chloroplast genome assembly. Complete chloroplast genome sequences were constructed by matching and linking contigs [81] and filling the gaps after assembly using second-generation sequencing technology.

The chloroplast genomes of the four *Swertia* L. species were annotated using the online program GeSeq [82] and PGA software [83]. We compared annotations from the two methods and made final adjustments manually in Geneious version 11.0.2 [81]. Then, we checked the initial annotation and putative starts, stops, and intron positions by comparison with homologous genes in congeneric species *S. mussotii*. Then, we used OGDRAW [84] software to draw circular plastid genome maps of the four *Swertia* L. species. Finally, the sequence data and gene annotation information of the four *Swertia* L. species were uploaded to the NCBI database with accession numbers NC_056357 (*S. franchetiana*), ON164641 (*S. przewalskii*), ON017794 (*S. tetraptera*), and SUB11740174 (*S. bifolia*).

### Sequence alignment

Including the 30 previously sequenced plastomes in GenBank, plastome sequences (using both coding and noncoding regions) from 34 species in Subtribe Swertiinae were aligned with MAFFT (version 7) [85] using the default settings. The plastomes of subtribe Swertiinae are conserved in gene order and content, so the alignment was straightforward; some poorly aligned regions were manually adjusted in GENEIOUSv.8.1 [81]. Different methods for handling gaps have a certain impact on the final results [86]. Therefore, it is very important to address the gaps in the data set when data are analyzed. As shown by previous research results, reliable gaps are within comparison areas of high confidence, showing a kind of indel event during the sequence evolution process. Therefore, reliable gaps also reflect evolutionary history of sequences, and should be treated as characters, just as bases. Unreliable gaps are in fuzzy comparison areas and categorized as sites of uncertain alignment. They are excluded from systematic analysis and not included in the analyzed data set. In this research, unreliable gaps were deleted from the compared sequences before analysis.

### Single sequence repeat (SSR) and relative synonymous sodon usage (RSCU) analysis

We used the online MISA program [87] to detect SSRs in the chloroplast genomes of 34 species in subtribe Swertiinae using the following parameters: mononucleotide unit repetition number ≥ 10; dinucleotide unit repetition number ≥ 5; trinucleotide unit repetition number ≥ 4; and tetranucleotide, pentanucleotide, and hexanucleotide unit repetition number ≥ 3 [87]. CodonW1.4.2 software was also employed to confirm the amino acid usage frequency and relative synonymous codon usage (RSCU) [88].

### Comparative analysis of complete chloroplast genome

We used IRscope software to visually analyze boundaries between the four main chloroplast regions (LSC/IRb/SSC/IRa) of 34 species in subtribe Swertiinae [89]. Moreover, Mauve software was used to analyze the chloroplast DNA rearrangement of the 34 species in subtribe Swertiinae. Meanwhile, the online software mVISTA was used to compare the 34 species of Subtribe Swertiinae in shuffle-LAGAN Mode [90]. The genome of *V. Baillonii* was used as the reference genome. The method developed by Zhang et al. (2011) [91] was used to calculate the percentages of variable characters in the coding and noncoding regions of the chloroplast genomes. The proportion of mutation events = [(NS + ID)/L] × 100, where NS = the number of nucleotide substitutions, ID = the number of indels, and L = the aligned sequence length.

### Analysis of synonymous (Ks) and non-synonymous (Ka) substitution rate

We computed the selective pressures for protein-encoding genes that were located in three regions of the chloroplast genomes (LSC, SSC and one IR). Protein-coding genes that were shared by the 34 species were chosen and extracted from complete chloroplast genomes for synonymous (*K*s) and nonsynonymous (*K*a) substitution rate analysis. Each gene selection was forecast by taking into account the ratios of *Ka*/*Ks*, that is, *Ka*/*Ks* < 1 purifying selection, *Ka*/*Ks* = 1 neutral selection, and *Ka*/*Ks* > 1 positive selection [92]. Nonsynonymous (*K*a) and synonymous (*K*s) substitution rates were calculated using KaKs_Calculator 2.0 software [93] with the following settings: genetic code table 11 (bacterial and plant plastid code) and method of calculation: NG.

### Phylogenetic analysis

To examine the phylogenetic relationship of 34 species of 9 genera within subtribe Swertiinae, an evolutionary tree was constructed with the complete chloroplast DNA sequences using *G. straminea* Maxim. (KJ657732), *Gardneria ovata* Wall. (NC_065470) and *Amalocalyx microlobus* Pierre (NC_067035) as outgroups. Meanwhile, we used 80 shared protein-coding genes of 34 chloroplast genomes to construct a molecular phylogenetic tree. Phylogenetic analyses were performed according to the Bayesian inference (BI) method under the best-fit substitution model GTR + I + G selected by the AIC in MrModeltest 2.3 [94] using MrBayes v3.2.1 [95]. BI analysis was run independently using four Markov Chain Monte Carlo (MCMC) chains, that is, three heated chains and one cold chain, and started with a random tree; each chain was run for 2 × 10^7^ generations and sampled every 2 000 generations, and discarding the first 25% preheated trees as burn-in. We estimated the convergence of data runs using an average standard deviation of split frequencies (ASDSF) < 0.01 and Tracer v1.7.1 [96] to check for an effective sample size (ESS) > 200. The phylogenetic tree nodes were considered well-supported when the node Bayesian posterior probability (BP) was ≥ 0.95.

### Divergence-time estimation

The divergence times of subtribe Swertiinae species was estimated using BEAST v1.8.4 software package [97]. The chloroplast genomes sequences from 34 species of subtribe Swertiinae and outgroups were included in the analysis. Firstly, BEAUti in the software package was used to set the parameters of nexus format sequence file. The optimal nucleotide substitution model selected by MrModeltest was GTR + I + G. An uncorrelated relaxed clock analysis with a Yule process speciation model was specified. Pollen of *Lisianthius* P. Browne (Trib. Potalieae, Gentianaceae) from Eocene sediments in Panama was used as lognormal priors, with an offset at 33.6 Ma [98], a mean of 0.7, and a standard deviation of 1.0, as used by Matuszak et al. (2015) [99]. The final Markov chain was run seven for 10 million generations, sampling every 10,00 generations. After the parameters were set, the BEAST file in xml format was generated. Secondly, the XML format file was imported into the BEAST software. Thirdly, Tracer v1.7.1 [96] was used to assess the convergence and stability of run parameters, i.e., ESS of parameters > 200. Fourthly, Maximum clade credibility (MCC) trees were produced using TreeAnnotator v2.4.1 [100] by applying a burn-in (as trees) of 10%, posterior probability limit of 0.5, and median height for node selection. Finally, FigTree v1.4.4 [101] was used to edit and visualize the time tree.

## Supplementary Information

**Additional file 1:** **Table S1.** Genes content of 34 species in 9 genera from Subtribe Swertiinae.

**Additional file 2:** **Table S2.** Length of genes with introns in the cp genomes of 34 species of 9 genus in Subtribe Swertiinae.

**Additional file 3:** **Table S3.**  SSR analysis for the cp genome of 34 species in 9 genus of Subtribe Swertiinae.

**Additional file 4:** **Table S4.** Relative synonymous codon usage statistics.

**Additional file 5:** **Table S5.** The GenBankaccession numbers of Subtribe Swertiinae species complete chloroplast genomesthat were downloaded from GenBank

**Additional file 6: Figure S1. **Phylogenetic tree of 34Subtribe Swertiinaespecies using Bayesian inference (BI) analysesbased on protein-codinggene sequences. Numbers at nodes correspond to Bayesian inference (BI) posterior probabilities (only probabilities ≥0.95 are shown).

## Data Availability

All data generated or analyzed during this study are included in this published article and its supplementary information files. The datasets generated and/or analyzed during the current study are available in the GenBank repository (*S. przewalskii*: https://www.ncbi.nlm.nih.gov/nuccore/ON164641; *S. franchetiana*: https://www.ncbi.nlm.nih.gov/nuccore/NC_056357.1/; *S. tetraptera*: https://www.ncbi.nlm.nih.gov/nuccore/ON017794; S. *bifolia*: SUB11740174).
