# Supplementary material for: Comparative chloroplast genomics of 34 species in subtribe Swertiinae (Gentianaceae) with implications for its phylogeny
Source: BMC Plant Biol. 2023 Mar 28;23:164. doi: 10.1186/s12870-023-04183-1 (PMC10044379; doi:10.1186/s12870-023-04183-1)
Supplement: Supplementary file 5 — Additional file 5: Table S5. The GenBankaccession numbers of Subtribe Swertiinae species complete chloroplast genomesthat were downloaded from GenBank [file 12870_2023_4183_MOESM5_ESM.docx]

Table S5 The GenBank accession numbers of Subtribe Swertiinae species complete chloroplast genomes that were downloaded from GenBank

| Species | GenBank accession numbers |
| --- | --- |
| *Comastoma falcatum* | MK331815 |
| *Comastoma pulmonarium* | MW324577 |
| *Gentianopsis barbata* | MZ579704 |
| *Gentianopsis grandis* | NC_049879 |
| *Gentianopsis paludosa* | MT921831 |
| *Lomatogoniopsis alpina* | NC_050658 |
| *Lomatogonium perenne* | NC_050659 |
| *Pterygocalyx volubilis* | NC_056992 |
| *Veratrilla baillonii* | MW872006 |
| *Halenia coreana* | MK606372 |
| *Halenia elliptica* | NC_050657 |
| *Swertia bimaculata* | MW344296 |
| *Swertia cincta* | MZ261898 |
| *Swertia cordata* | NC_054359 |
| *Swertia dichotoma* | MZ261899.1 |
| *Swertia dilatata* | MW344298 |
| *Swertia diluta* | NC_057681.1 |
| *Swertia erythrosticta* | MW344299 |
| *Swertia franchetiana* | NC_056357 |
| *Swertia hispidicalyx* | NC_044474 |
| *Swertia kouitchensis* | MZ261902 |
| *Swertia leducii* | NC_045301 |
| *Swertia macrosperma* | MZ261903 |
| *Swertia multicaulis* | NC_050660 |
| *Swertia nervosa* | NC_057596 |
| *Swertia pubescens* | MZ261905 |
| *Swertia punicea* | MZ261896 |
| *Swertia souliei* | NC_052874 |
| *Swertia verticillifolia* | MF795137 |
| *Swertia wolfgangiana* | MW344307 |
